# Perceived human factors from the perspective of paramedics – a qualitative interview study

**DOI:** 10.1186/s12873-022-00738-x

**Published:** 2022-11-11

**Authors:** Anna Poranen, Anne Kouvonen, Hilla Nordquist

**Affiliations:** 1 Faculty of Medicine, University of Helsinki, 00014 Helsinki, Finland; 2 Faculty of Social Sciences, University of Helsinki, 00014 Helsinki, Finland; 3 Centre for Public Health, Queen’s University Belfast, BT12 6BA Belfast, Northern Ireland; 4 South-Eastern Finland University of Applied Sciences, 48220 Kotka, Finland

**Keywords:** Emergency medical services, Ergonomics, Paramedic, Work environment, Prehospital emergency care

## Abstract

**Background:**

The work environment in prehospital emergency medical care setting is dynamic and complex and includes many stressors. However, little is known about the perceived human factors from the perspective of paramedics. In this study, we investigated, from the perspective of paramedics, what are the human factors, and how are they linked to prehospital emergency medical care?

**Methods:**

Data were collected through semi-structured interviews (*n* = 15) with Finnish paramedics. The material was analyzed using inductive content analysis.

**Results:**

Three main categories of human factors were identified. The first main category consisted of factors related to work which were divided into two generic categories: “Challenging organizational work environment” and “Changing external work environment.” The second main category comprised factors related to paramedics themselves and were divided into three generic categories: “Issues linked to personality,” “Personal experiences”, and “Factors resulting from personal features.” The third main category described that paramedics have difficulties in understanding and describing human factors.

**Conclusion:**

This study revealed numerous factors that can affect paramedics’ work in the EMS setting. Increased knowledge about human factors in the EMS setting provides organizations with the opportunity to develop procedures that can support paramedics’ cognitive and physical work. Human factors in different situations can be addressed to improve occupational and patient safety*.*

**Supplementary Information:**

The online version contains supplementary material available at 10.1186/s12873-022-00738-x.

## Background

Since *To Err Is Human* was published in 1999 [1] increasing attention has been paid to human factors in health care. It is now better understood that human action is valuable and essential because it withstands variability [2]. However, humans are fallible and perfect human action is impossible especially in stressful and demanding environments [3]. Human factors are not causes of errors in the action of individuals. Human factors are all the features and functions that are related to work environment, organizations, teams, individuals or work itself [4, 5], and interact with human behavior [4, 6]. Human factors focus on the interactions between humans and the work environment; and they can both support and hinder operation and safety [4, 5]. By understanding human factors, systems and procedures can be designed in such a way that humans’ performance can be supported [6].

Although health care systems and procedures have been developed to support the work of health care professionals, the prehospital emergency medical care setting has not received the same amount of attention as the hospital setting [7]. The work environment associated with emergency medical services (EMS) is highly complex and challenging and contains numerous stressors [8, 9]. Paramedics frequently face unexpected, sudden situations in their line of work, and they are expected to perform in any circumstance or environment under continuous time pressure. Time pressure has been demonstrated to be a significant stress factor, and a feeling of being rushed in one’s work has been reported to be as one of the human factors in the EMS setting [10–13]. Stressful situations can reduce the cognitive processes of the paramedics and affect their decision-making, situational awareness, and problem-solving abilities [14–16]. Furthermore, shift work, fatigue and other physiological limitations cause additional challenges to working in EMS [8, 17–19].

Misasi & Keebler [7] have described the importance of investigating human factors and stated that EMS is “one of the most cognitively and physically demanding jobs in health care”. A gap in the literature regarding human factors in EMS was pointed out in a systematic review by Bigham et al. [20]. Aspects that are linked to human factors in EMS setting have not been identified specifically from paramedics’ point of view. It is crucial to better understand the human factors in the EMS setting to ensure occupational and patient safety as well as to improve paramedics’ well-being and performance [21]. Therefore, the present study investigated the human factors in the prehospital emergency medical care setting. To this end, the following research question was formulated: from the perspective of paramedics, what are the human factors, and how are they linked to prehospital emergency medical care?

## Methods

### Design

A qualitative interview design was used in this study because human factors have scarcely been studied in the context of prehospital emergency medical care. The consolidated criteria for reporting qualitative research (COREQ) checklist were used to ensure the quality of reporting this study [22] (Supplementary file 1).

### Setting

This study was carried out in Finland where EMS is organized by hospital districts, in co-operation with local rescue services or by outsourcing the services to the private sector [23]. The Finnish EMS consist of advanced level and basic level ambulances. In addition, every hospital district has always at least one field supervisor on duty.

Advanced-level EMS units are staffed by at least one bachelor’s degree level paramedic and one other qualified person, for instance, a health care professional or a firefighter. Advanced-level paramedics are at least registered nurses (3.5 years training) with additional advanced life support education or prehospital nurses (4 years training). Basic-level EMS units are staffed by one health care professional (a vocational upper secondary qualification) with specialization in prehospital emergency care (comparable to emergency medical technicians or basic life support level) and one other qualified person. Field supervisors (responsible of operational work) are advanced-level paramedics with sufficient work experience and an operative leadership training [24, 25].

In Finland, EMS is a part of healthcare services and specifically, they complement the services of emergency medical department. The main role of EMS in Finland is to treat critically ill patients who have an injury or a sudden onset of severe illness. However, during the last decades their role has changed, and EMS has developed to treat patients more often in patients’ homes. Paramedics assess the need of care, referring patients to appropriate health or social care services, and planning patient’s care with other health and social care professionals [23, 26, 27].

### Participants

Participating paramedics were recruited via social media. Specifically, inclusion criteria were that paramedics were from an advanced or basic levels, or field supervisors with any length of experience, and at the time of recruitment they were currently working in EMS in different EMS areas in Finland. Participants were invited from a Finnish Facebook group “Ensihoidon uutiset” (in English: News of Prehospital Emergency Medical Services) which has over 5000 members working in EMS settings across Finland. The administrator of the Facebook group was contacted, and their permission was sought to publishing a recruitment ad in June 2020. Potential participants were asked to contact the first author via Facebook Messenger and then confirm their participation via email. All interested paramedics were included in the study. Participants received more information about the study upon contacting the first author and before they confirmed their participation.

Eighteen paramedics contacted the first author, but two of them did not confirm their participation and one paramedic had hoped for a different data collection method. Finally, fifteen paramedics confirmed their participation and were included in this study. Participant group consisted of advanced-level paramedics and field supervisors who represented seven EMS in Finland and included both men (*n* = 6) and women (*n* = 9).

### Data collection

The study data were collected via semi-structured individual interviews as these types of interviews enable dialogue between the interviewer and participant [28]. The first and the last author formulated the interview questions and topics based on theoretical information and the gaps in the literature. Before the interviews, an external expert on human factors assessed the suitability of the questions and themes. A pilot interview with a potential study participant was organized, and the interview questions were amended based on the expert’s comments and the pilot interview. The interviews began with the question, “What do human factors mean to you?” Subsequent questions encouraged the participants to describe the issues that, according to them, are linked to human factors in the EMS setting. While a few follow-up questions were predesigned, most of them were formed based on the participants’ earlier responses. For instance, a follow-up question was, “You said that tiredness is a human factor, could you describe how it is linked to your work in EMS?”

Interviews were carried out in person (*n* = 8), online using Skype or Microsoft Teams (*n* = 4) and by phone (*n* = 3) between July and October 2020. Each interview lasted between 40 and 75 minutes and were conducted in Finnish. All the interviews were audio recorded by one external device and transcribed by the first author. The transcriptions were assigned with numerical codes and pseudonymized so that participants cannot be identified. No personal information was transcribed, and confidentiality of identity was guaranteed throughout the research process. The study material was stored on a safe university network drive in a password protected folder that could only be accessed by the authors.

### Ethical considerations

Participation in the study was voluntary. All participants were informed about the study purpose, process, and that the interview responses would be used for scientific purposes. A statement of data protection and information about researchers (females) were shared before interviews. Following this, each participant signed a consent form. In the phone and online interviews, a signed consent form was emailed back to the first author. The study protocol was reviewed and approved by South-Eastern Finland University of Applied Sciences ethics committee on May 19, 2020. The ethical principles and good scientific practices defined by the Finnish National Board on Research Integrity [29] were followed throughout the research process.

### Data analysis

Inductive content analysis [30] was used in this study. First, the transcripts were read several times by the first author in order to get familiar with the content and understand the participants’ perceptions. After that, short sentences were chosen as units of analysis. Many participants described their perceptions in broad terms, due to which short sentences were chosen contextually represent the responses a context in the later phases of the analysis. All the participants’ perceptions were coded by the first author and without the use of any analysis software.

Following this, the codes were collated into a chart. The codes were summarized in plain form, and any overlaps were removed. The abstracted codes were then divided into subcategories which were grouped into upper categories and generic categories. Finally, main categories were formed based on the generic categories [30]. Category grouping was jointly performed by the first and the last author.

### Reflexivity statement

Interviews were conducted by the first author, an advanced-level paramedic with 7 years of experience in EMS. A clinical experience in the EMS setting was beneficial when asking specific follow-up questions during the semi-structured interviews and to gain a deeper understanding of the participants’ perceptions. On the other hand, the interviewer’s preunderstanding of the research topic may cause an unidentified, non-excludable lack of openness toward the subject. During the interviews, an open and trusting dialogue was held to ensure that the interviewer is not going to impact participant’s responses. The interviewer was not more highly placed compared to participants. The study was carefully designed, and the analysis was collaboratively performed by the first and the last author to ensure that participants’ perceptions were interpret correctly. The last author has a several years of academic research and supervisory experience in the EMS setting, and a comprehensive understanding of the research method used. Finalization of the formed categories was done in collaboration between the first, second, and the last authors. The second author has an extensive experience regarding academic research, methodology and supervision, and a wide understanding of occupational health and related social phenomena.

## Results

**Fig. 1:**
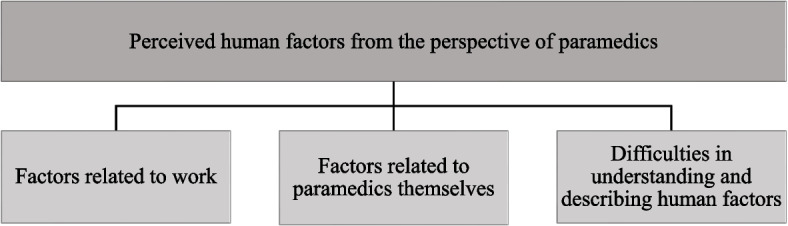
Main categories of the perceived human factors from the perspective of paramedics

Perceived human factors from the perspective of paramedics formed three main categories: 1) factors related to work; 2) factors related to paramedics themselves; and 3) difficulties in understanding and describing human factors. An overview of the categories is displayed in Supplementary file 2. Fig. 1 provides an overview of the main categories.

### Factors related to work

**Fig. 2:**
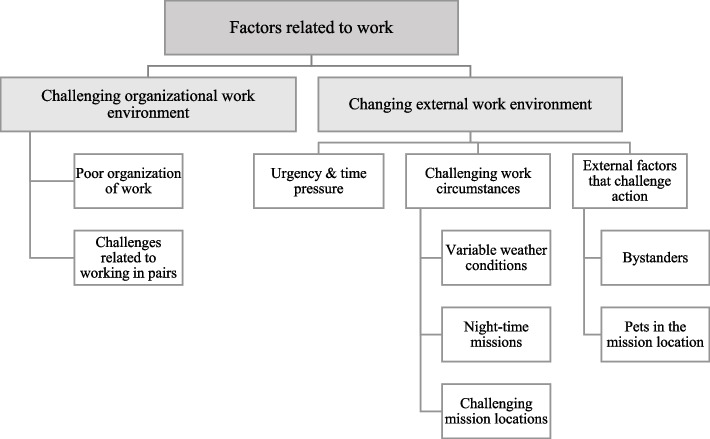
An overview of the main category “Factors related to work”

The first main category “Factors related to work” was divided into two generic categories: “Challenging organizational work environment” and “Changing external work environment”. Fig. 2 provides an overview of the first main category.

#### Challenging organizational work environment

The category “Challenging organizational work environment” was divided into two upper categories: “Poor organization of work” and “Challenges related to working in pairs”.

##### Poor organization of work

The participants stated that some of the existing standard operating procedures (SOPs) are not easily accessible and that there are excessive protocols in place; these factors negatively affect paramedical missions. Lack of certain equipment was also considered a human factor because it might affect the procedures undertaken during missions.

*When we don’t have certain equipment, we are forced to deviate from the desired procedure. (P13)*

##### Challenges related to working in pairs

The participants reported that the interpersonal relationship between work partners is a human factor in EMS settings. If work partners do not get along with each other, information may not be transmitted adequately between them.

*If the interpersonal relationship between work partners is not adequate, information may not be transmitted. This is a really big risk factor for both work and patient safety. (P1)*

The different roles in a team and the clarity of said roles and operating models determined in advance were also found to be factors that can affect pair work. In addition, the expectations and assumptions related to working in a team or in pairs and trusting one’s work partners were reported as human factors in the interviews. However, excessively strong trust was seen as an operational risk if a paramedic who is overly confident in their partner’s manner of working may not observe their partner’s action, leading to something essential going unnoticed. The functionality of co-operation between the work partners in a pair or team was introduced as a human factor, as were communication and situational awareness.

*The working methods of the partners can never be too clear because such a scenario would only lead to one partner not taking care to oversee the other’s work or result in issues being assumed, which is very risky – tasks may remain undone, or each partner may do something without knowing what the other has been done. (P4)*

#### Changing external work environment

The category “Changing external work environment” was divided into three upper categories: “Urgency and time pressure,” “Challenging work circumstances”, and “External factors that challenge action.”

##### Urgency and time pressure

The participants stated that urgency is a factor that poses challenges in prehospital emergency medical care because time pressure can affect a paramedic’s performance or behavior. Urgency and time pressure were seen as causing a paramedic to accidentally omit a task or unknowingly make mistakes. Urgency was pointed out as a risk factor for control over the entire mission, especially, when a paramedic must hurry and manage multiple issues at the same time.

*You feel hurried, when you’re given a time frame within which to manage a mission; this kind of time pressure might lead to changes in your behavior, or you might forget to do something. (P7)*

##### Challenging work circumstances

The participants conveyed that different weather conditions pose a risk for paramedics and patients at the scenes in EMS settings. Icy surfaces and freezing weather were particularly mentioned in the interviews. The impossibility of affecting weather conditions was mentioned as a human factor.

*The courtyard was totally icy and slippery. In addition, it was freezing outside, and we paramedics were both freezing, and the patient was freezing even more, so the situation was filled with delays. (P12)*

The participants said that mission time is one of the human factors in prehospital emergency medical care. Specifically, night-time missions were described as having an impact on the working capacity and behaviors of paramedics. For instance, it was noted that situations causing request for clarification by the patients or bystanders or dissatisfaction with patient care typically occurred at night.

*I have often noticed, when managing requests for clarification from patients, their family members, or bystanders, these situations occur in the evening or at night. (P6)*

Mission location was another human factor conveyed by the participants. For instance, a paramedic would find it challenging to carry out their work if the patient may be in a cramped place. They may even be assigned a missions that is on an island, resulting in additional travel time by boat. Wrong addresses also increase time delays and challenge paramedics’ work.

*Is the patient, for example, in a cramped place? Such a place would make work difficult. (P15)*

##### External factors that challenge action

Various external factors may cause challenges managing a mission. For instance, when paramedics are called to patient’s home, the patient’s family members may impede or disturb their work which is exacerbated by the fact that family members cannot be removed from the situation (in their own home) unlike in an institutional setting.

*At a hospital, you can ask the nurses or security staff to take the family members aside, but in prehospital situations, you cannot remove family members from their own home involuntarily. (P13)*

The presence of pets at an emergency scene can present another challenge for the paramedics. Paramedics may not have prepared themselves for handling pets beforehand, and it may not be possible to remove pets from the situation.

*It may be that an animal is in the scene. We cannot inevitably prepare for animals and when a patient does not want to remove, for example, a big dog from the situation, that can cause difficulties. (P10)*

### Factors related to paramedics themselves

The second main category “Factors related to paramedics themselves” was divided into three generic categories: “Issues linked to personality,” “Personal experiences”, and “Factors resulting from personal features.” In general, the participants identified paramedics as central to human factors because many individual aspects can affect human action.

*Broadly speaking, a paramedic himself is at center. (P8)*

**Fig. 3:**
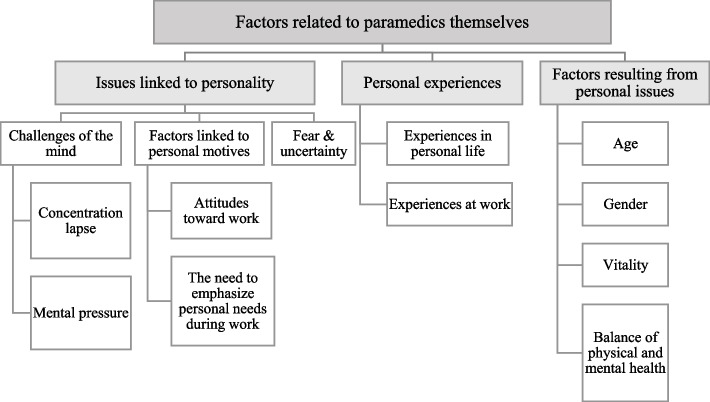
An overview of the main category “Factors related to paramedics themselves”

An overview of the main category “Factors related to paramedics themselves” is given in Fig. 3.

#### Issues linked to personality

The category “Issues linked to personality” was divided into three subcategories: “Challenges of mind,” “Factors linked to personal motives” and “Fear and uncertainty.”

##### Challenges of the mind

The participants conveyed that it is more difficult to concentrate on paramedical mission if one’s thoughts are elsewhere. The participants stated that it would be difficult to keep one’s focus on a mission after returning to work from a holiday, for instance, which is especially risky if the first mission is critical. In addition, a paramedic who takes an extra shift, may find it difficult to maintain their concentration and orientation.

*When you go on holiday, you typically reset your mind. So, reorientating yourself to work takes time. Plus, if your shift starts at seven o’clock and you are leaving for a difficult mission right away, then you will surely not be in the same mood as you were before your holiday. (P1)*

According to the participants, paramedics’ inattention to managing a mission may lead to misunderstandings and unintended thoughtlessness, making this a human factor as well. Events that take place during the work shift can lead to concentration lapses which may affect the management of subsequent missions if a paramedic remains fixated on past events. A challenging mission would require a lot of work and cognitive resources, and a paramedic’s stress tolerance is linked to keeping focus in that situation. Perception and cognitive processes were also seen as human factors by the participants.

*Simply put, your thoughts are somewhere else, then you might do something that should not be done or that must be done in a different way. (P11)*

Mental pressure and mental overload were seen as human factors by the participants.

*Mental fatigue … if you were on a mission that shocked you or made you go off the rails, that will have an impact on how you manage the next mission. (P1)*

A paramedic’s work experience can also affect how they manage the mental load during prehospital emergency medical care missions. Missions that are mentally shocking tend to be memorable and cause mental load and pressure. New, inexperienced paramedics who retrospectively evaluate their work may experience increased stress, which affect their ability to manage subsequent missions. The interviews revealed that new paramedics worry about others thinking they do not have the required competence to work in EMS.

*That mental workload … Are you an experienced or inexperienced paramedic, and how will you manage mental factors during your shift? (P8)*

##### Factors linked to personal motives

Attitudes toward work and work motivation were described as human factors by the participants. Motivation affects one’s management of work tasks. According to the participants, a paramedic’s weak motivation may lead to one-sided thinking. Motivation was also linked to vigilance and the management of work tasks.

*Irrespective of whether the task is small or big, a motivated paramedic would think the task through more thoroughly and with more viewpoints, showing determination in the way the task is managed, than a paramedic who is cutting corners (P14).*

Based on the interviews, frustration is a human factor in prehospital emergency medical care. The paramedics described their frustration and poor attitudes toward non-urgent missions when having to handle an increased number of missions overall. Having the wrong attitude may cause a paramedic’s poor orientation toward patient care. For instance, if the transport time to the hospital is short, it is easier to pick up the patient without familiarizing with their issues. Lack of motivation may adversely affect the patient’s treatment.

Attitudes are ‘f**king s**t’ toward unhurried missions and, actually, toward other missions as well. They are decided based on the emergency call information, the task code or if the patient is already known … That kind of attitude. There is no hero work for paramedics at the scene. (P3)

Additional SOPs and numerous changes to existing SOPs can also increase paramedics’ frustration. According to the participants, they find it difficult to internalize new changes and to train themselves according to new SOPs. If their attitudes toward modified and new SOPs are poor, paramedics are unable to maintain their skills and easily slip back to following the old procedures.

*There are a lot of changes all the time, and there are people who don’t want to keep up with the changes and want to stick to the old rules. When everything is changing so quickly, paramedics are resistant to them and don’t have the interest or persistence to keep up or read about new protocols. (P3)*

The need to emphasize personal physical needs of paramedics during missions was also highlighted. According to the participants, when a mission takes place at the end of the paramedic’s shift, it is managed in a more straightforward way because the paramedic would wish to return home at the earliest. A paramedic’s levels of food and rest can also affect a patient’s care. Thus, hunger was determined to be a human factor, as decreased blood sugar adversely affects paramedics’ clarity of thought.

*Hunger … if blood sugar is low, then perhaps you will hurry to complete the work, not consciously intending to bungle it up. But you think you might be able to get out of the situation faster and have something to eat … or if you did not have time to take a break, or you may have the need to go to the toilet. (P12)*

##### Fear and uncertainty

Paramedics may unconsciously react their fear of something. According to the participants, fear may cause paramedics to rush to get out of a scene if they think it is not safe. This may cause paramedics to forget or be afraid to do something essential. Fear of errors or failure was also mentioned in the interviews. If a paramedic is afraid that they might fail, their actions may lead to errors.

*You think you must get out quickly, so something essential is not done or you are afraid of doing it. There is fear. (P11)*

According to the participants, uncertainty about one’s own work and skills can affect a paramedic’s actions in prehospital emergency medical care missions. If work partners are of different levels, the less experienced paramedic may feel that the greater experience of the other paramedic adds pressure to missions; this is compounded by the former’s lack of courage to reveal their uncertainty and ask for help.

*There’s uncertainty, but you are afraid to show it. So, feelings … feelings are human factors. (P7)*

#### Personal experiences

This category consisted of two subcategories: “Experiences in personal life” and “Experiences at work.”

##### Experiences in personal life

A paramedic’s personal life situation and personal experiences influence how they interact with patients during prehospital emergency medical care. Personal experiences of similar situations may challenge a paramedic’s ability to manage their own feelings and thoughts. However, those experiences can also enable the paramedic to approach the situation in a more humanly and empathetic manner. According to the participants, personal issues may remain at the forefront of one’s mind and distract them from their work. Personal crises, such as, divorce or financial problems, can reduce a paramedic’s focus on patient treatment.

*Situations at home can affect you a lot because, if something is going badly at home, your thoughts are focused on that situation and not on the mission. (P1)*

##### Experiences at work

According to the participants, paramedics may better understand causations and process issues if they have previously experienced a similar situation. Paramedics can reflect on and adapt solutions that work in the past to the current situation. However, various factors may affect the current situation, which makes mirroring of solutions difficult. Furthermore, some paramedics base their work on the existing facts whereas others base their work, often unconsciously, on their own thoughts and experiences instead of the facts. Accordingly, certain missions may be clearer in paramedic’s mind and affect how they manage the following missions.

*A paramedic may use the same model for managing a current situation, but the new situation may be totally different and have other factors that affect it. (P2)*

#### Factors resulting from personal features

This upper category was divided on four subcategories: “Age,” “Gender,” “Vitality”, and “Balance of physical and mental health.”

##### Age

According to the participants, younger paramedics may search for solutions more actively and quickly during missions. Further, younger paramedics may think that only two options – one good solution and one bad one – are available in any situation, and they may not apply their theoretical knowledge when making decisions. In contrast, older paramedics are more likely to understand that there are many options in a situation, with greater number of bad ones. According to the participants, age brings with it varied perceptions and experiences as well as cognitive skills. Learning skills will also change.

*When you are older, you see that there are many options; nothing’s black and white. There are also more bad options. (P2)*

##### Gender

It was suggested that cognitive features differ between women and men, in turn affecting work. For example, it was stated that if the physical features of the two genders are different, the cognitive features would be too.

*A man and a woman are different in their physical features, so they are also different in their cognitive features, which relate to gender. (P2).*

##### Vitality

The participants suggested that vitality (tiredness versus alertness) is one of the human factors associated with prehospital emergency medical care, as it was seen to affect paramedics’ performance and behaviors at work. An alert paramedic tends to have higher resilience, and the decisions made vary accordingly. The participants also stated that a paramedic’s orientation toward a mission when they are woken up for on-call duty work can be considered a human factor.

A tired paramedic may pay attention to irrelevant issues during a patient interview. Tiredness also makes emergency response driving risky, especially for long distances at night. Fatigue can decrease one’s evaluation skills, attention and motivation. According to the participants, fatigue makes work more automatic. When a paramedic carries out procedures automatically; for instance, going about the ingrained motions without conscious thought, deviant measurements may go unnoticed and essential questions may go unasked. Physical fatigue is caused by, for example, a high number of missions during a work shift.

*You do not necessarily understand everything [when you are tired]. You automatically write down the numerical values of measurements, but you don’t understand when you’re tired that the value is a little low or a little high. You do not notice those kinds of numerical issues, and you also don’t remember to ask everything. (P3)*

##### Balance of physical and mental health

According to the participants, balance between the physical and mental health of a paramedic is linked to the paramedic’s work in EMS and is therefore a human factor. Recovery from long work shifts was pointed out to be a notable factor affecting paramedics’ physical and mental health. It was mentioned that exhaustion and burnout can increase paramedics’ disregard of their work in the long run. An exhausted paramedic does not have the strength to care about whether the work is of good quality and follows safety protocols.

*The worker’s own balance of physical and mental health affects their work. (P8)*

### Difficulties in understanding and describing human factors

Participants also found it challenging to describe their opinions about human factors and the issues associated with human factors in prehospital emergency medical care. According to the participants, human factors are issues that affect the technical performance and fulfilment of work tasks and impact paramedics’ performance. Almost all participants mentioned that human factors are directly linked to human errors.

*It causes … or it affects the way we manage work or do tasks, such that errors are bound to happen. (P11)*

According to some of the participants, paramedics cannot influence human factors and human factors are things which humans are performing right or wrong by mistake. Human factors involve coincidence, for instance, some issues never happen again in the same way. Some participants stated that the concept of human factors is challenging to comprehend, and that they have difficulties concretely identifying human factors in practice.

*Human factors are a little talked about concept and people do not ponder over it … Let’s take health care, for instance, we are humans, yes, but what does that mean in practice? … This is a difficult concept, I think. (P5)*

## Discussion

This study investigated the perceived human factors from the perspective of paramedics. In the analysis, human factors were divided into three main categories. The first category comprised factors related to work. The second category consisted of factors related to paramedics themselves. The third category described the issue of human factors being conceptually difficult to grasp.

The results showed that according to paramedics a number of aspects related to the work environment tend to impact in their work. Previous studies have similarly demonstrated that unexpected situations and working around the clock in challenging and even dangerous environments can increase paramedics’ stress levels and pose occupational and patient safety risks [9]. In this study, urgency and time pressure were described to be human factors that affect the behavior and performance of paramedics. These results are in line with previous studies [10–12].

A lack of clarity and poor availability of SOPs was also considered as a human factor that can affect paramedics’ performance, cause frustration, and decrease work motivation. These results are consistent with previous findings that a lack of guidelines increases the activation of unconscious and fast intuitive information processing. This kind of information processing is based on experiences and feelings, due to which it may be affected by cognitive biases [31–33]. Frustration caused by unclear guidelines was also mentioned in Fairbanks et al.’s [34] study.

A good interpersonal relationship between work partners was described to be an important human factor in the EMS setting. This result is in line with a previous study, which found that communication between team members can fail if cooperation is not ensured [31]. In earlier studies, paramedics’ personal braveness and fear of criticism were shown to affect the working atmosphere within a team and diminish the utilization of all available knowledge and resources of team members [34]. This was confirmed in the present study. Overall, the current findings show that according to paramedics, human factors are not only individual-level issues but are also important at the organizational level. Thus, it is important for organizations to become aware of these issues and understand the importance of developing SOPs and paying attention to teamwork.

Besides the challenging nature of the work environment, the present results demonstrate that several aspects linked to paramedics themselves can be felt as human factors. Paramedics may experience mental overload and pressure if unpleasant memories of past events remain at the forefront of their minds. Similar to these findings, previous studies have demonstrated that patients’ acute crises can cause stress in paramedics [20], and that critical situations can cause strong emotional reactions [35]. In addition, present study’s findings show that paramedics’ personal motives, such as their attitudes toward work, and personal needs during missions are experienced as human factors in EMS settings. Work motivation was linked to the management of work tasks and to vigilance, which can affect patient safety. Frustration was linked to the quality of patient care, especially in non-urgent missions. To our knowledge, similar results related to paramedics’ work motivation and attitudes have not been previously demonstrated. However, Stefurak et al. [36] showed that work motivation and job satisfaction are necessary and should be prioritized because the EMS work environment is complex, and the work is highly demanding.

The results further show that paramedics link previous personal and work experiences to human factors. Past work experiences can affect an individual’s approach to information processing, and experienced paramedics can apply their knowledge from previous experiences to new situations. This not only shows the importance of analytical information processing, especially in the case of non-urgent missions, but also that of intuitive information processing to find solutions. These results are in line with the Andersson Hagiwara et al.’s [37] report that thinking involves a combination of analytical and intuitive information processing and that paramedics utilize previous experiences in the decision-making process. What was surprising is that inexperienced paramedics may feel mental stress if their work partner is highly experienced. This has not been previously demonstrated.

In the present study, the participants mentioned vitality and fatigue as human factors that significantly influence the management of work tasks. These factors were further linked to various mission-related aspects such as evaluation ability, concentration and vigilance. There are many studies that have investigated vitality and fatigue in EMS setting and how they affect, for instance, patient safety, performance at work and emergency response driving [11, 18, 19, 38, 39]. The findings of vitality and fatigue in this study are in line with existing literature.

Overall, the issues related to paramedics themselves clearly show how different factors can impact paramedical work and human action. Establishing supporting procedures and checklists can help paramedics systematically address all the essential aspects of any mission and reduce the variability of function, thus improving patient safety. Checklists, for instance, may reduce the effects of stress and fatigue and help standardize patient treatment. Checklists could make paramedics’ work more systematic, and treatments can be done with a more structured way, thus the quality and safety of care can be improved [40, 41].

Notably, many of the interviewed paramedics stated that human factors can be difficult to identify. Moreover, they relayed that the conceptualization of human factors is incomprehensible on a practical level and that human factors are directly linked to human errors. Furthermore, participants described issues which in literature are not directly linked to human factors such as harsh weather conditions. That finding shows that human factors are not entirely understood in practical level and these findings also support the ideas of Russ et al. [6], who stated that the very concept of human factors may be misleading and cause misunderstandings. In addition, human factors are widely presented as causing failures and that is why a common misconception of human factors hampers the development of health care.

### Limitations

The sample size for the present study was small, which may be a limitation. Participation was voluntary and, therefore, self-selected. There was a sign of saturation after fourteen interviews as no additional aspects were mentioned during the fifteenth interview. However, two additional participants could have been recruited to ensure that data saturation was achieved. Furthermore, the results revealed that the research subject was at least partly unfamiliar to some paramedics, which might have affected their willingness to participate in the study.

Participants did not get any description of human factors prior to, or in the beginning of interviews, which may be a limitation. It is thus possible that participants did not describe all aspects that are related to human factors but on the other hand, they mentioned some new issues that have not been previously recognized as human factors in the literature.

The participants were from across Finland which brings diversity to the sample and allowed obtaining data from different EMS operating areas. Although the locations or EMS operating areas were not the focus of the data analysis, it can be seen that paramedics from different EMS operating areas face similar challenges. Furthermore, there is a possibility that paramedics’ perceptions regarding human factors may differ based on their work experience in EMS, but this information was not collected in the present study. The lack of background information is a result of the recruitment letter in which the participants were assured that no personal information is recorded. The qualitative approach allowed this, as the results aim to describe the overall phenomenon. Social media offers an efficient way to recruit participants but only paramedics who use Facebook could be reached and it might affect the number of potential participants.

Most of the interviews were carried out in-person but a few were conducted remotely. Online meetings offer more flexibility; for example, it is easier to recruit participants from geographically larger areas [42]. Phone interviews may produce different results because interactions tend to be more challenging without nonverbal cues. In the present study, a rapport was maintained during the phone interviews, and a good dialogue between the interviewer and the participant was established.

## Conclusion

The present study gives an insight to perceived human factors from the perspective of paramedics in the EMS setting in Finland. Overall, this study strengthens our research understanding of human factors at the individual and organizational levels. Taken together, it can be inferred from our results that the factors that affect paramedics’ work are linked to the nature and organization of work and work environment.

The findings have several important implications for future practice. Increased knowledge about human factors in the EMS setting provides organizations with the opportunity to develop procedures that can support paramedics’ cognitive and physical work. Thus, human factors in different situations can be addressed to improve occupational and patient safety*.*

A natural next step of research in this area would be a larger and more diverse study examining human factors in EMS. In addition, further progression of research in this area would involve analyzing and determining the types of procedures and tools that can support paramedics in their work. Future research could also explore the factors that affect decision-making processes and thinking in depth by observing, for example, simulated situations.

## Supplementary Information

**Additional file 1: **COREQ (COnsolidated criteria for REporting Qualitative research) Checklist.

**Additional file 2.**

## Data Availability

The datasets generated and analysed during the current study are not publicly available due the ethical reasons but are available from the corresponding author on reasonable request. The informed consent contained a statement that only researchers have access to the raw data and that findings would be presented in an anonymised way.

## Abbreviations

EMS: Emergency medical services
SOP: Standard operating procedure
