# Supplementary material for: Perceived human factors from the perspective of paramedics – a qualitative interview study
Source: BMC Emerg Med. 2022 Nov 11;22:178. doi: 10.1186/s12873-022-00738-x (PMC9652815; doi:10.1186/s12873-022-00738-x)
Supplement: Supplementary file 2 — Additional file 2. [file 12873_2022_738_MOESM2_ESM.docx]

**Supplementary file 2.**
